# Seagrass-Derived Cellulose/Collagen Composite Coating for Enhanced Tomato Shelf Life and Postharvest Quality

**DOI:** 10.3390/polym18010076

**Published:** 2025-12-26

**Authors:** Senthilkumar Palanisamy, Kokila Saravanan, Jishna Jabbar, Rahul Jacob Michael, Barani Kumar Saravana Kumar, Jintae Lee, Devaraj Bharathi

**Affiliations:** 1 Sabanci University Nanotechnology Research and Application Center (SUNUM), Istanbul 34956, Turkey; 2 School of Biotechnology, Dr. G R Damodaran College of Science, Coimbatore 641014, India; 23mbtkokilasaravanan@grd.edu.in (K.S.); 23mbtjishnaj@grd.edu.in (J.J.); 23mbtrahuljacobm@grd.edu.in (R.J.M.); 23bbtbaranikumarsaravanakumar@grd.edu.in (B.K.S.K.); 3 School of Chemical Engineering, Yeungnam University, 280 Daehak-Ro, Gyeongsan 38541, Republic of Korea; jtlee@ynu.ac.kr

**Keywords:** seagrass cellulose, collagen, tomato shelf life, antifungal activity, sustainable packaging

## Abstract

This study explored an eco-friendly coating system combining seagrass-derived cellulose fiber (SCF) from *Cymodocea rotundata* with marine type I collagen (MC) for tomato preservation. The SCF/MC composite was prepared through enzymatic and natural crosslinking processes and subsequently characterized using X-ray diffraction (XRD), Fourier transform infrared spectroscopy (FTIR), field emission scanning electron microscopy (FESEM), and thermogravimetric analysis (TGA). The results demonstrated that SCF/MC possessed a compact morphology, strong hydrogen bonding interactions, high crystallinity, and excellent thermal stability. When applied as a coating, SCF/MC composite significantly reduced weight loss in tomatoes, preserved firmness (>39 Units), regulated acidity, maintained moisture levels (~90%), and delayed increase in pH compared to the uncoated control. Additionally, the SCF/MC coating sustained ascorbic acid and moderated lycopene accumulation, indicating delayed ripening. At 0.5% of SCF/MC composite, spoilage was limited to 22% versus ~80% in control samples, demonstrating a substantial reduction in decay. Antifungal assay showed strong inhibition of *Aspergillus flavus*, with the highest suppression of mycelial growth observed at 0.5% of SCF/MC. Overall, the SCF/MC coating effectively enhanced fungal safety and maintained the physicochemical quality of tomatoes, thereby extending shelf life while valorizing seagrass biomass as a sustainable postharvest resource.

## 1. Introduction

Tomatoes (*Solanum lycopersicum*) are among the most widely consumed fruits worldwide and are valued for their nutritional profile, including vitamins, antioxidants, and dietary fiber [1]. However, postharvest losses at both farm and retail stages in India are estimated to range between 25% and 30%, primarily due to high moisture content of tomatoes, their low firmness, and most notably microbial spoilage [2,3]. Such losses highlight the need for sustainable preservation methods that can maintain fruit quality and extend shelf life [4].

Natural edible coatings are widely used postharvest preservation strategies and are increasingly formulated using natural biopolymers [5,6]. Among these coating materials, cellulose is particularly valuable due to its high mechanical strength, excellent film-forming ability, renewable origin, non-toxicity, thermal stability, and biodegradability [7,8]. When applied to fruit surfaces, cellulose forms a semipermeable barrier that reduces respiration, limits microbial intrusion, slows enzymatic browning, regulates gas exchange, and minimizes moisture loss, thereby extending shelf life [9,10]. Beyond its barrier function, cellulose also provides a stable structural matrix suitable for combining with compatible biopolymers to enhance coating performance [11]. In this study, marine type I collagen (MC) was chosen as a complementary component due to its strong water retention capacity, good film adhesion, and ability to reinforce mechanical stability through hydrogen bonding. Combining SCF with MC enables the formation of a compact, bioactive network with superior barrier and antifungal properties.

Marine seagrasses are progressively recognized as important reservoirs of bioactive constituents with significant potential for industrial applications [12]. Among these species, the tropical seagrass *Cymodocea rotundata*, which grows along the coasts of the Gulf of Mannar, has been reported to exhibit a wide variety of biological activities. The pharmacological potential associated with these bio-functional properties underscores the importance of investigating seagrass-derived metabolites for the sustainable development of therapeutic and nutraceutical products [13,14]. More importantly, the utilization of seagrass biomass for cellulose extraction aligns with waste-to-wealth paradigms, enabling the transformation of underutilized marine resources into high-value, environmentally benign biopolymers that deliver concurrent environmental and economic benefits.

Notably, no previous studies have reported the integration of cellulose isolated from *C. rotundata* with MC for postharvest applications, positioning this work as the first to develop a marine-derived dual-polymer edible coating exhibiting enhanced mechanical strength, barrier performance, and antifungal properties. In this study, a liquid edible coating system was selected as dip-coating enables uniform surface coverage, strong adhesion to tomato surfaces, and minimal material consumption, making it particularly suitable for postharvest preservation. To strengthen the coating matrix, genipin and microbial transglutaminase (TGase) were employed as natural and enzymatic crosslinkers, respectively. TGase promotes covalent crosslinking between collagen amino groups, while genipin stabilizes the network through additional crosslink formation, resulting in a compact, mechanically robust cellulose-collagen composite. Accordingly, the objective of this study was to produce a SCF/MC composite derived from *C. rotundata*, evaluate its efficacy in extending tomato shelf life, and assess its antifungal performance. The anticipated outcomes are expected to support the development of functional edible coatings for perishable produce while advancing the sustainable valorization of seagrass biomass within a waste-to-wealth framework.

## 2. Materials and Methods

### 2.1. Materials

A potential seagrass species, *C. rotundata,* was harvested during its active growth phase from Thondi (Lat. 9°45′ N, long. 79°30′ E) in the Gulf of Mannar, Tamil Nadu, India. Healthy leaves were selected and gently scraped with a sterile spatula to remove debris and epiphytic diatoms. The leaves were then washed with distilled water and brought to the lab for additional examination. The tomatoes were inspected visually to make sure they were uniform in size, color, and free of fungus after being bought at a local market in Coimbatore.

MC powder was procured from a commercial supplier (Mahatreya Herbals, Chennai, India, Good Manufacturing Practice (GMP) and ISO 9001:2015 certified). The product was non-GMO, gluten-free, and supplied as a fine powder suitable for biomedical applications. All other chemicals used in the study, including genipin (CAS no.: 6902-77-8), microbial transglutaminase (product no: SAE0159), ascorbic acid (PCT0207), and Sabouraud Dextrose Agar (SDA) (SMH063), were of analytical grade and were procured from Sigma-Aldrich and HiMedia, respectively.

### 2.2. Isolation of Cellulose Fiber from C. rotundata

The cellulose extraction procedure was adapted with slight modification from established cellulosic biomass pretreatment protocols reported by Qasim et al. [15]. To preserve structural integrity and reduce moisture content, freshly collected *C. rotundata* seagrass was initially shade-dried for two weeks. After thorough washing with distilled water to remove adhering salts, sand, and epiphytes, the dried biomass was oven-dried at 110 °C for 24 h in a hot-air oven. To soften tissues and facilitate the removal of the waxy outer layer, the dried seagrass fragments were immersed in hot distilled water (80 °C) for 3 h. The pretreated biomass was subjected to Soxhlet extraction for 24 h using a hexane-ethanol (2:1, *v*/*v*) to remove pigments, oils, and residual contaminants. Following delignification, the material was filtered and further purified using sodium bisulfite solution and deionized water. The pH was adjusted to neutral by the gradual addition of 10% (*v*/*v*) acetic acid. The suspension was subsequently subjected to five centrifugation cycles to remove residual impurities, and the recovered fibers were dried at 110 °C in a hot-air oven. The resulting raw cellulose was further purified by treatment with a 10:1 (*v*/*v*) mixture of 70% nitric acid and 80% acetic acid at 110 °C for 10 min, thereby enhancing cellulose purity. The purified cellulose fibers were filtered, extensively washed with deionized water, and oven-dried. The final cellulose product was stored in sterile, airtight containers until further use.

### 2.3. Preparation of SCF/MC Composite

The collagen solubilization and TGase/genipin crosslinking procedures were carried out as described by Rao et al. [16]. Marine collagen (1% *w*/*v*) was dissolved in 0.5 M acetic acid (pH 3.2) at 4 °C for 2 h under gentle stirring to obtain a transparent, homogeneous solution. Purified seagrass cellulose fibers (1% *w*/*v*) were dispersed separately in distilled water. The collagen and cellulose dispersion solutions were then combined and continuously agitated for 30 min at 25 °C to ensure homogeneous mixing. To enhance the mechanical strength and structural stability of the composite, microbial transglutaminase (TGase, 10 U g^−1^ protein) and genipin (0.5% *w*/*v*, pH 7.0) were introduced to enable enzymatic and natural crosslinking, respectively.

### 2.4. Characterization of SCF/MC Composite

XRD, FTIR, TGA, and FE-SEM analyses were performed following standard analytical procedures described by Rasheed et al. [17]. The crystalline structure of the cellulose fiber was examined using an XRD (X’Pert Pro diffractometer, PANalytical BV, Almelo, Netherlands) equipped with Cu/Kα radiation over a 2θ range of 10–80°. Functional groups were identified in the wavelength range of 4000–500 cm^−1^ using the FTIR (Shimadzu MIRacle 10, Shimadzu Corporation, Kyoto, Japan) with the KBr pellet method. Thermal characteristics and degradation behavior were evaluated by TGA (EXSTAR 6000 TG/DTA 6300 analyzer, Seiko Instruments Inc., Chiba, Japan) under a nitrogen environment at a heating rate of 10 °C/min^−1^ over a temperature range between 30 and 900 °C. For FE-SEM analysis, dried SCF/MC composite was cut into approximately 5 × 5 mm pieces and mounted on aluminum stubs using carbon tape. The samples were gold-sputtered to a thickness of 8–10 nm using Quorum Q150R ES sputter coater (Quorum Technologies Ltd., Lewes, UK) and examined using a FEI Quanta 200 FE-SEM (FEI Company, Hillsboro, OR, USA) at an accelerating voltage of 20 kV. Micrographs were acquired at 50, 40, 20, and 10 µm magnifications to assess surface morphology and cellulose-collagen interactions.

### 2.5. Effectiveness of SCF and SCF/MC Composite Treatment on Tomato Shelf Life and Physicochemical Characteristics

#### 2.5.1. Development of SCF/MC Composite Coating Solution

The procedures for determining physicochemical characteristics were adapted from the methods of Palanisamy et al. [18]. About 10 tomatoes were allocated to each treatment group: control, SCF alone, and SCF/MC composite at 0.2% and 0.5% (*w*/*v*). In this study, SCF/MC was prepared as a liquid coating suspension (emulsion-type dispersion), which formed a thin cellulose–collagen layer on the tomato surface after dipping and air-drying. The fruits were submerged for two minutes in a 0.05% NaOCl solution to ensure surface cleanliness. Tomatoes were dipped in the corresponding SCF or SCF/MC composite solutions (0.2% and 0.5%) for two minutes, while the control group was treated with distilled water. Coated fruits were kept at 25 ± 1 °C with 85% relative humidity and allowed to air-dry for a short period of time in order to evaluate physiological parameters and shelf-life.

#### 2.5.2. Evaluation of Weight Loss, Moisture, and Firmness

To calculate the weight loss during storage, the initial weight of each tomato was noted, and its weight at various intervals was compared. The percentage of weight loss was calculated using (1):

Weight loss (%) = [(Initial weight − Final weight)/Initial weight] × 100 (1)

The moisture content was determined using a modified oven drying method based on the Association of Official Analytical Chemists (AOAC, 2000), as described by Horwitz [19]. Five tomatoes from each group were homogenized and transferred into pre-weighed moisture dishes. Samples were dried in a hot-air oven at 105 °C for 24 h, cooled in a desiccator for 30 min, and reweighed. Moisture content (%) was calculated as (2):

Moisture content (%) = [(Initial weight − Final weight)/Initial weight] × 100 (2)

Fruit firmness was measured using a LABART penetrometer fruit hardness tester GY-3, with ten fruits tested per treatment, and measurements taken at three equidistant points along the equatorial region of each fruit.

#### 2.5.3. Assessment of Titratable Acidity and pH

A digital pH meter (HM Digital, Mumbai, India) calibrated with standard buffer solutions of pH 4.0 and 7.0 was used to measure the pH of tomato juice samples collected from control, SCF, and SCF/MC (0.2% and 0.5%) treated fruits. During the measurement, a magnetic stirrer was used to constantly spin around 10 mL of juice in a beaker at 25 ± 0.5 °C. Ten grams of each tomato sample were mixed with 30 mL of distilled water, filtered, and the filtrate was diluted to 100 mL in order to determine the titratable acidity (TA). The results were stated as a percentage of citric acid after a 10 mL aliquot of the diluted juice was titrated against 0.1 N NaOH using phenolphthalein as an indicator.

#### 2.5.4. Determination of Lycopene and Ascorbic Acid

To measure ascorbic acid, 5 g of tomato pulp from each treatment was homogenized using a T25 digital ULTRA-TURRAX homogenizer (IKA-Werke GmbH & Co. KG, Staufen, Germany) with 5 mL of 1% hydrochloric acid. The absorbance of the clear supernatant was measured at 243 nm after the homogenate was centrifuged for 10 min at 10,000 rpm. A standard L-ascorbic acid calibration curve was used to determine the ascorbic acid concentration, and the results were reported as mg of ascorbic acid per 100 g of fresh weight (mg/100 g FW). About 5 g of tomato tissue was homogenized with 50 mL of an acetone–hexane combination (3:5, *v*/*v*) and centrifuged at 5000 rpm for 10 min in order to analyze lycopene. Using the proper extinction coefficient, the amount of lycopene was determined by measuring the absorbance of the upper phase at 645 nm. The amount of lycopene was measured in milligrams per 100 g of fresh weight (mg/100 g FW).

#### 2.5.5. Determination of Decay Percentage

Tomato fruits were visually inspected for indications of spoiling, such as fungal growth, softness, or discoloration, in order to evaluate tomato degradation during storage. Using the following Formula (3), the decay % was determined:

Decay (%) = Number of decayed fruits/Total number of fruits × 100 (3)

To track the effectiveness of the coatings in lowering postharvest degradation, tomatoes coated with control, SCF, and SCF/MC composite were evaluated at regular intervals during the storage period.

### 2.6. Antifungal Activity of SCF, and SCF/MC

The antifungal assay was followed by the modified method of Palanisamy et al. [20]. The antifungal activity of SCF and SCF/MC against *A. flavus* was evaluated using mycelial growth inhibition on SDA supplemented with each composite at a final concentration of 10 µg/mL. Spore were collected from a 7-day-old culture using 0.1% Tween-80, and a standardized suspension was prepared by adjusting the concentration to 1 × 10^2^ spores/mL. To ensure uniform inoculum density, 10 µL of the spore suspension was placed at the center of each SCF or SCF/MC composite-enriched agar plate. The plates were incubated at 30 °C for 15 days. After incubation, the radialgrowth of the fungal colony was measured from the center outward, and the colony radius was recorded. Uncoated plates served as negative controls. Antifungal inhibition was calculated using Formula (4).

Inhibition (%) = [(Growth of control − Growth of treatment)/Growth of control] × 100 (4)

### 2.7. Statistical Analysis

All experiments were performed in triplicate (n = 3) of tomatoes treated with SCF, SCF/MC, and the untreated control, and results were expressed as mean ± standard deviation. Statistical analyses were performed using SPSS software (version 30; SPSS Inc., Cary, NC, USA). One-way analysis of variance (ANOVA) was applied to determine significant differences among treatments. Post hoc comparisons were conducted using Duncan’s Multiple Range Test (DMRT) at a significance level of *p* < 0.05. For all measured parameters, *p*-values, error bars, and replicate data (n = 3) were included in the analysis.

## 3. Results and Discussion

### 3.1. Formation of SCF and SCF/MC Composite

**Figure 1:**
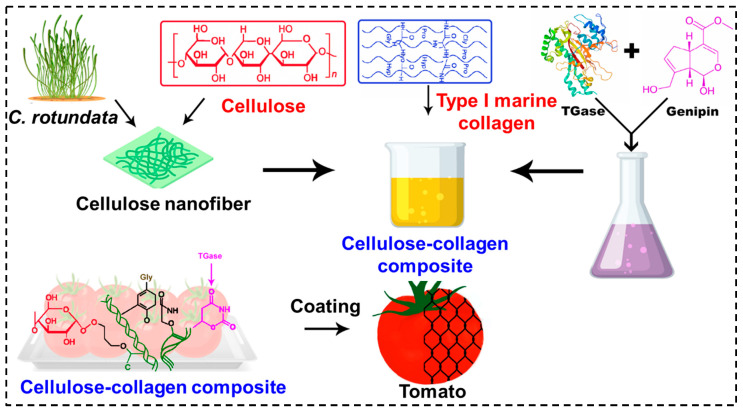
Schematic illustration of seagrass cellulose–collagen coating preparation. *C. rotundata* cellulose was mixed with MC and crosslinked with genipin and TGase to create a stable composite for tomato shelf-life improvement.

Isolation and purification of cellulose fibers from *C. rotundata* through the selective elimination of non-cellulosic components and associated contaminants yielded about 43% of the initial dry weight of the seagrass biomass (Figure S1). The purified cellulose fibers were subsequently combined with MC to produce a composite material, using TGase and genipin as enzymatic and natural crosslinking agents, respectively. TGase facilitated the formation of covalent bonds between collagen amino groups and cellulose hydroxyl groups, while genipin further stabilized the polymeric network through reactions with collagen amino groups, resulting in the formation of stable intermolecular crosslinks [21]. This dual crosslinking strategy significantly enhanced the mechanical stability, intermolecular interactions, and antifungal properties of the SCF/MC composite, making it especially effective as a coating on tomatoes to promote shelf life, physiological quality, and inhibit fungal growth (Figure 1) [22].

### 3.2. Characterization of Prepared SCF and SCF/MC

**Figure 2:**
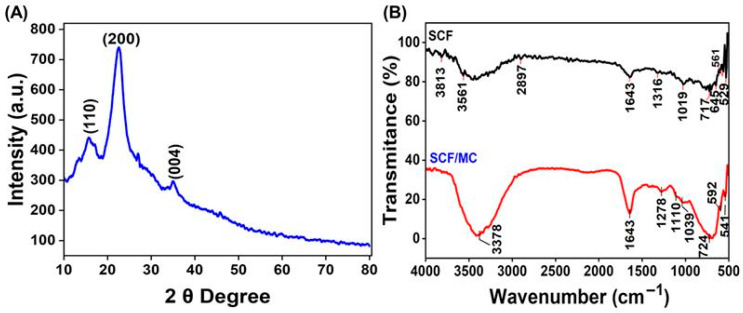
(**A**) XRD pattern of the cellulose obtained from *C. rotundata*. (**B**) FTIR spectra of SCF and SCF/MC composite.

XRD analysis confirmed the crystalline structure of the cellulose derived from *C. rotundata* (Figure 2A). Characteristic peaks of cellulose I were obtained at 2θ ≈ 16° (110), 22° (200), and 34° (004) [23,24]. Although smaller peaks denote less ordered planes, the dominant (200) reflection showed the presence of distinct crystalline areas. According to Park et al. [25] and Kljun et al. [26], the sharp (200) peak indicates high crystallinity, which adds to improved tensile strength, toughness, and thermal stability essential for coating applications. Pretreatment provided improved crystallinity by efficiently eliminating lignin and hemicellulose [26]. After TGase- and genipin-mediated crosslinking, this structural integrity improves collagen compatibility and yields subsequent stable cellulose-collagen composites appropriate for food preservation [21].

The FTIR spectra of SCF and SCF/MC composite show characteristic changes associated with collagen incorporation (Figure 2B). In raw SCF, the broad band at 3561 cm^−1^–3813 cm^−1^ corresponds to O-H stretching vibrations of cellulose [27]. This band shifts to 3378 cm^−1^ in the SCF/MC composite, indicating enhanced hydrogen bonding between cellulose hydroxyl groups and collagen’s amide functionalities. The typical cellulose C-H stretching peak at 2897 cm^−1^ is retained, confirming preservation of the polysaccharide backbone. The SCF/MC composite shows a more pronounced amide I band at 1643 cm^−1^, associated with C=O stretching of collagen, which is weaker in pure SCF, confirming collagen incorporation [18]. The peaks at 1316 and 1278 cm^−1^ (amide III, C-N stretching) and 1110–1039 cm^−1^ (C-O-C and C-O stretching of cellulose) suggest overlapping vibrations from both components [28]. In the lower wavenumber region, changes in peaks such as 724, 592, and 541 cm^−1^ indicate modifications in the fingerprint region due to molecular interactions and structural rearrangement with cellulose without disrupting the major cellulose structural framework. The characteristic collagen-associated amide I and II peaks present in the SCF/MC spectrum clearly confirm the successful incorporation of collagen into the SCF/MC composite and are thus expected to increase its mechanical strength, structural stability, and biocompatibility [29,30].

**Figure 3:**
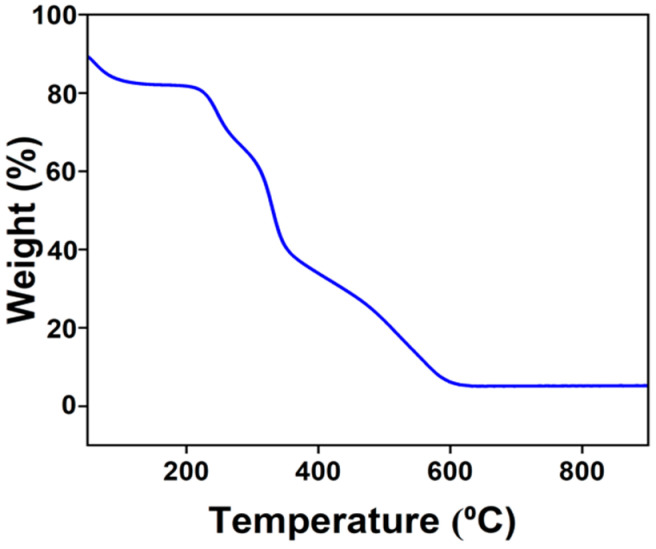
TGA thermogram of the SCF/MC composite highlighting thermal stability and degradation behavior.

TGA of the SCF/MC composite under nitrogen revealed a multistage thermal degradation profile (Figure 3). The initial weight loss of ~8.8% below 200 °C corresponded to the evaporation of physically adsorbed and bound water, with an estimated Tonset of approximately 175 °C [31]. The major degradation event occurred between 350 and 500 °C, with a Tonset of approximately 400 °C and a Tmax near 500 °C, representing crystalline cellulose depolymerization and breakdown of the collagen peptide backbone [32]. A further ~24.6% weight reduction between 500 and 650 °C reflected degradation of more thermally stable crosslinked residues. Beyond 650 °C, the curve plateaued with a residual mass of ~10%, attributed to thermally stable inorganic ash components naturally present in seagrass-derived cellulose [33].

**Figure 4:**
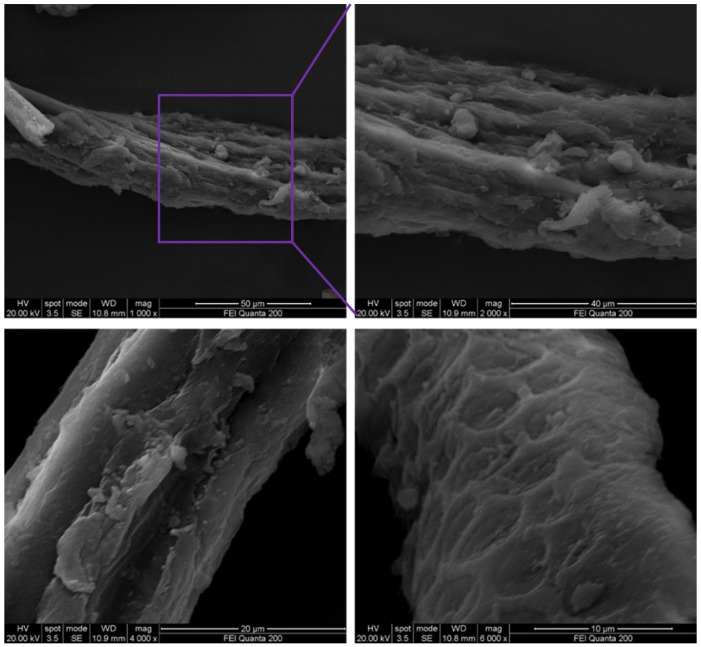
FESEM micrographs showing the surface morphology and cellulose–collagen interactions of the SCF/MC composite at 50, 40, 20, and 10 µm magnifications.

FESEM analysis of the SCF/MC composite revealed characteristic microstructural features resulting from collagen incorporation (Figure 4). The composite displayed a denser and more uniform surface morphology compared to raw fibers, indicating improved structural packing within the matrix rather than directly observable cellulose–collagen interactions. Although gas permeability and coating thickness were not evaluated in this study, the compact and homogeneous surface arrangement suggests the potential for reduced gas and moisture transmission, as frequently reported for cellulose–collagen composite systems [34,35].

### 3.3. The Effect of SCF and SCF/MC Treatment on the Physicochemical Properties and Shelf Life of Tomatoes

**Figure 5:**
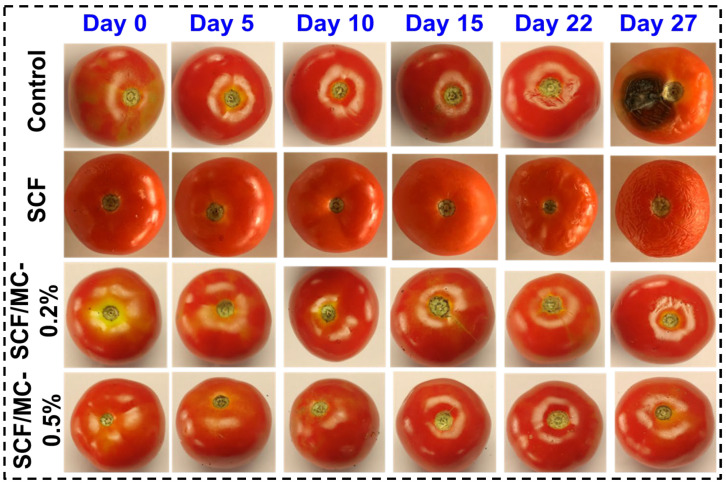
Visual appearance of tomatoes coated with different treatments (control, SCF, SCF/MC–0.2%, and SCF/MC–0.5%) during storage for up to 27 days.

Visual assessment revealed marked differences in the shelf life of tomatoes among the treatments and control samples (Figure 5). The tomatoes that had the SCF-coating showed an ability to shrink but a slower onset of spoilage, while tomatoes that did not have the SCF-coating rotted by day 22 and decomposed by day 27. The SCF/MC coating was found to be more effective; the 0.2% concentration minimized shrinkage to day 22, whereas the 0.5% coating was effective in preserving the freshness and inhibited fungal growth to day 27. This enhanced activity is attributed to stronger cellulose–collagen crosslinking facilitated by TGase and genipin [36], along with the semipermeable barrier effect that reduces respiration and microbial invasion [37]. Subsequently, the SCF/MC 0.5% coating provided the ideal result, extending shelf life to 27 days.

**Figure 6:**
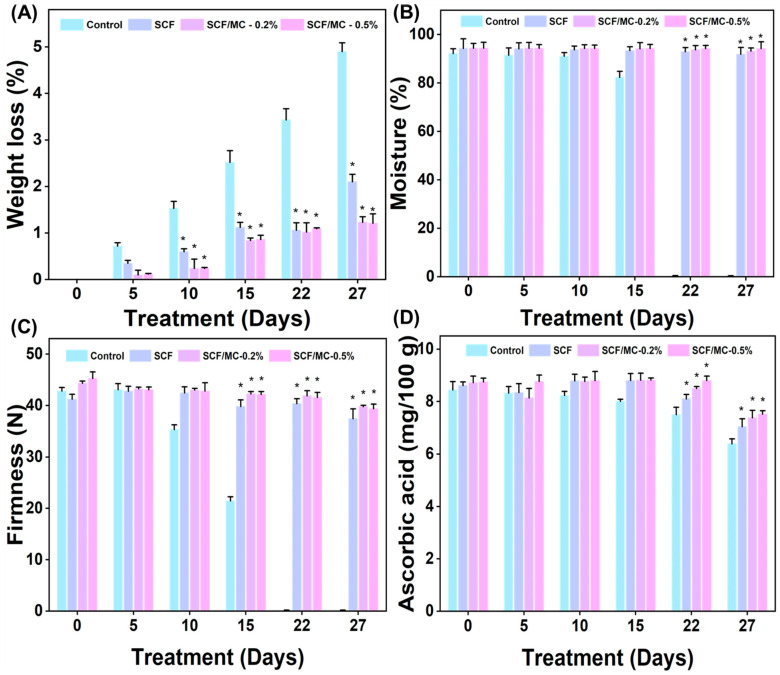
The effect of SCF and SCF/MC (0.2% and 0.5%) coatings on tomato quality over a 27-day treatment period. (**A**) The weight loss percentage, (**B**) moisture content, (**C**) firmness, and (**D**) ascorbic acid content of treated and untreated tomatoes. Significant differences from the control (*p* < 0.05) are indicated by asterisks (*).

SCF and SCF/MC-coated tomatoes were evaluated for increased quality utilizing various physicochemical tests (Figure 6). By day 27, controls had rotted since they had lost about 5% of their weight (Figure 6A), almost 92% of their moisture content (Figure 6B), and a considerable amount of stiffness by day 22 (Figure 6C). SCF coatings exhibited decreased rigidity and shriveling, but deterioration was slowed. SCF/MC coatings were more successful in maintaining quality: 0.2% decreased weight loss to about 1.2% while preserving moisture and firmness, whereas 0.5% was the most effective, limiting weight loss to less than 2% while preserving around 90% moisture and firmness above 39 units on day 27. By reinforcing the matrix, collagen improves smoothness and water retention, while the semipermeable barrier lowers respiration and water loss [38].

**Figure 7:**
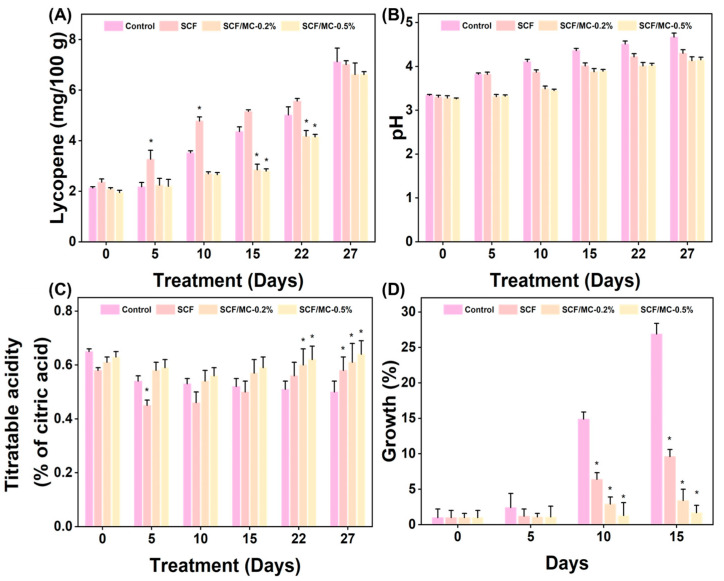
The effects of SCF and SCF/MC coatings on the biochemical characteristics of tomato after 27 days of treatment. (**A**) Lycopene content, (**B**) pH, (**C**) titratable acidity, and (**D**) *A. flavus* growth percentage of treated and untreated tomatoes. Significant differences from the control (*p* < 0.05) are indicated by asterisks (*).

The levels of ascorbic acid in coated samples were greater compared to those in the control group, and they decreased less after storage (Figure 6D). By day 27, SCF and SCF/MC were retaining 8.8–8.9 mg/100 g, while controls had 7.6 mg/100 g. These results indicate that barrier effects decreased oxidative losses [39]. Lycopene gradually increased across treatments (Figure 7A); SCF-coated fruits reached 7 mg/100 g, showing enhanced pigment development, while controls reached 7.1 mg/100 g, and SCF/MC coatings slowed accumulation (6.62 mg/100 g) due to delayed ripening under modified internal atmospheres [40]. SCF/MC coatings moderately delayed lycopene accumulation compared to the control.

The pH of SCF/MC (0.5%)-coated fruits remained within the range of 4.0 to 4.1 throughout storage, whereas the pH of control fruits exceeded to 4.67 by day 27, indicating reduced respiration rates and delayed ripening in the coated samples (Figure 7B) [41]. Although pH differences were modest, coated fruits consistently exhibited lower pH values than controls. The TA initially declined and subsequently increased during storage. Control fruits showed pronounced fluctuations in TA, whereas SCF treatment mitigated these changes. In contrast, SCF/MC (0.5%) effectively maintained a more stable TA throughout the storage period (Figure 7C). The slight recovery in titratable acidity during later storage may be attributed to reduced metabolic consumption of organic acids due to the semipermeable nature of the coating.

Tomato decay was decreased by SCF coatings to 40%, whereas control fruits had 80% decay by day 27 (Table 1). Performance was increased by adding collagen; SCF/MC 0.2% and 0.5% limited decay to 28% and 22%, respectively. Significant differences between treatments were observed (*p* < 0.05), most likely as a result of improved barrier and antibacterial capabilities, which is in line with previous research [42].

### 3.4. Antifungal Activity of SCF and SCF/MC Composite

SCF/MC coatings significantly inhibited *A. flavus* compared with the control and SCF alone treatments (Figure 7D). The fungal growth appeared in the control fruits by day 5 and rapidly progressed, resulting in extensive surface colonization by day 15. Although SCF coating delayed the onset of infection, fungal growth increased markedly during storage. In contrast, SCF/MC composite coatings suppressed fungal growth in a clear dose-dependent manner, with the 0.5% SCF/MC formulation providing the highest level of protection throughout the storage period (15 days), while the 0.2% formulation effectively restricted mycelial spread up to 5 days. This enhanced antifungal performance is likely due to the formation of a semipermeable cellulose-collagen barrier that limits nutrient availability, oxygen diffusion, and surface moisture [43,44]. Similar to our findings, gelatin-based films integrated with dialdehyde carboxymethyl cellulose and coffee leaf extract showed antibacterial activity against *S. aureus* and *E. coli* [45]. Another study by Pramesti et al. [46] reported that the collagen extracted from the sea cucumber exhibited antifungal activity against *Candida albicans*. SCF/MC coatings inhibit *A. flavus* by forming a dense, semipermeable cellulose-collagen barrier that limits oxygen, moisture, and nutrient diffusion, suppressing spore germination and hyphal growth [47,48]. The compact matrix enhances adhesion and restricts fungal colonization. Synergistic interactions between cellulose and collagen may have strengthened the composite coating, thereby prolonging antifungal protection [49,50].

## 4. Conclusions

Tomato preservation was significantly improved by SCF/MC composite coatings, as evidenced by reduced weight loss, improved firmness and moisture retention, preserved acidity, and effective inhibition of *A. flavus* growth. Among the tested formulations, the 0.5% SCF/MC coating demonstrated the highest efficacy, extending shelf life up to 27 days while maintaining superior quality features. These improvements are attributed to the synergistic barrier properties and stable crosslinked network formed between cellulose and collagen, further reinforced by genipin and TGase.

This study offers the first experimental evidence that marine collagen and cellulose isolated from seagrass can be combined to fabricate an environmentally benign edible coating for postharvest applications. While the coating components are food-grade, comprehensive evaluations of residue formation, leaching behavior, biodegradability under real storage conditions, sensory attributes, and biosafety were beyond the scope of the present work. Future studies should address these aspects, along with scalability and application to other perishable fruits and vegetables, to fully validate commercial feasibility and edible certification. Overall, SCF/MC coatings represent a promising marine-based, biodegradable alternative to synthetic postharvest treatments, supporting sustainable food packaging and preservation strategies.

## Figures and Tables

**Table 1:** Tomatoes treated with or without SCF, SCF/MC composite (0.2% and 0.5%), and their percentage of decay over 0, 5, 10, 15, 22, and 27 days of storage. Significant differences between treatments and the control at each time point are shown by different letters (*p* < 0.05).

Treatment	Decay (%)					
	Days of Storage					
	0	5	10	15	22	27
Control	0	5.2 ± 0.9 ^a^	15.1 ± 1.1 ^a^	30.3 ± 2.0 ^a^	55.4 ± 2.4 ^a^	80.1 ± 3.0 ^a^
SCF	0	3.1 ± 0.7 ^b^	8.7 ± 1.0 ^b^	15.5 ± 1.6 ^b^	28.1 ± 2.0 ^b^	40.5 ± 2.7 ^b^
SCF/MC-0.2%	0	2.0 ± 0.6 ^b,c^	5.3 ± 0.9 ^c^	10.2 ± 1.5 ^c^	19.9 ± 1.8 ^c^	28.4 ± 2.2 ^c^
SCF/MC-0.5%	0	1.5 ± 0.6 ^c^	4.1 ± 0.8 ^c^	8.1 ± 1.3 ^c^	16.2 ± 1.5 ^c^	22.1 ± 1.8 ^c^

## Data Availability

The original contributions presented in the study are included in the article and the Supplementary Materials; further inquiries can be directed to the corresponding authors.

## Supplementary Materials

The following supporting information can be downloaded at https://www.mdpi.com/article/10.3390/polym18010076/s1. Figure S1. Process of cellulose isolation from seagrass.
