# Supplementary material for: Seagrass-Derived Cellulose/Collagen Composite Coating for Enhanced Tomato Shelf Life and Postharvest Quality
Source: Polymers (Basel). 2025 Dec 26;18(1):76. doi: 10.3390/polym18010076 (PMC12787690; doi:10.3390/polym18010076)
Supplement: Supplementary file 1 [file polymers-18-00076-s001.zip › polymers-3996145-supplementary.pdf]

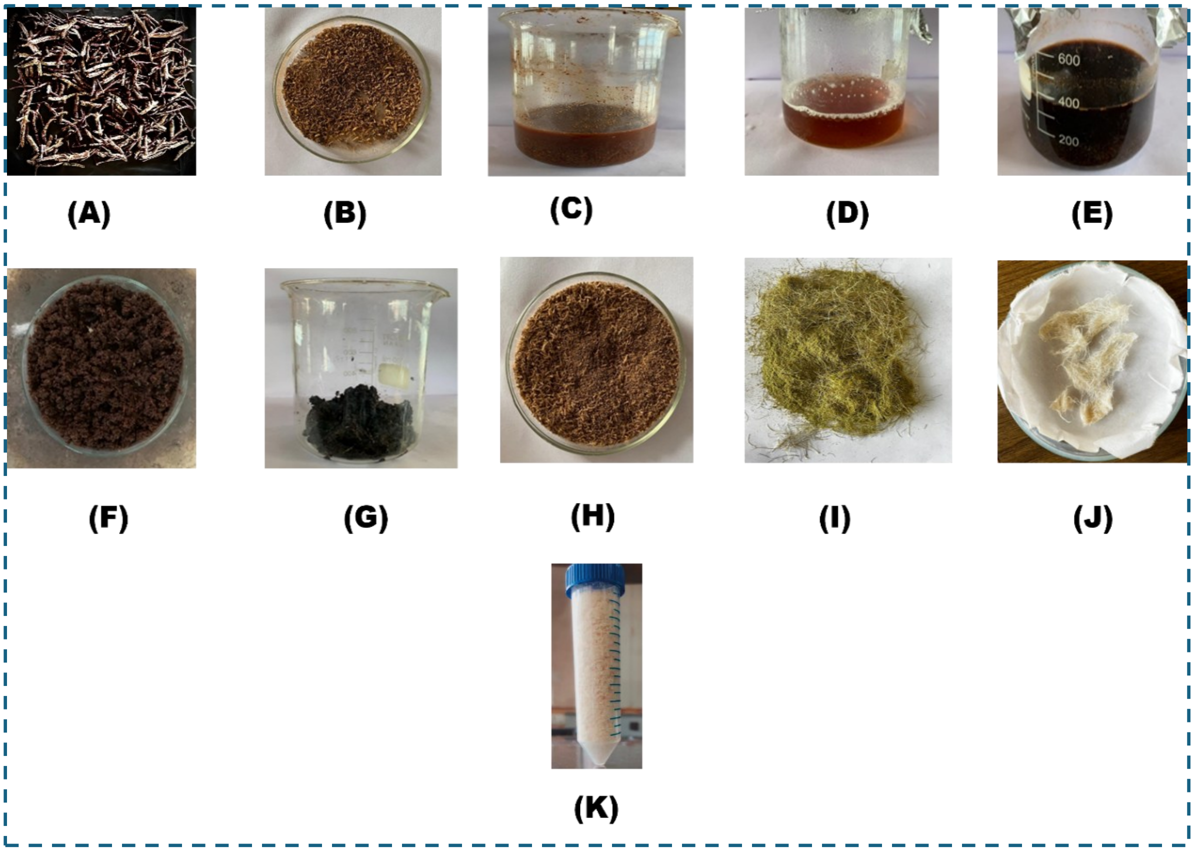

Figure S1. Isolation of cellulose fiber from seagrass. (A) Raw seagrass material. (B) Ground seagrass powder. (C) Alkaline treatment indicating lignin elimination. (D) Intermediate purification phase. (E) Concentrated alkali solution during treatment. (F) Alkali-treated residue after lignin extraction. (G) Bleaching treatment showing removal of residual impurities. (H) Purified cellulose powder. (I) Dried cellulose fibers. (J) Final isolated cellulose product. (K) Cellulose suspension demonstrating uniform nanofiber dispersion.
